# Activated Ras Signaling Pathways and Reovirus Oncolysis: An Update on the Mechanism of Preferential Reovirus Replication in Cancer Cells

**DOI:** 10.3389/fonc.2014.00167

**Published:** 2014-06-26

**Authors:** Jun Gong, Monica M. Mita

**Affiliations:** ^1^Department of Medicine, Cedars-Sinai Medical Center, Los Angeles, CA, USA; ^2^Samuel Oschin Comprehensive Cancer Institute, Cedars-Sinai Medical Center, Los Angeles, CA, USA

**Keywords:** reovirus, oncolysis, Ras, EGFR, PKR, apoptosis, necrosis, autophagy

## Abstract

The development of wild-type, unmodified Type 3 Dearing strain reovirus as an anticancer agent has currently expanded to 32 clinical trials (both completed and ongoing) involving reovirus in the treatment of cancer. It has been more than 30 years since the potential of reovirus as an anticancer agent was first identified in studies that demonstrated the preferential replication of reovirus in transformed cell lines but not in normal cells. Later investigations have revealed the involvement of activated Ras signaling pathways (both upstream and downstream) and key steps of the reovirus infectious cycle in promoting preferential replication in cancer cells with reovirus-induced cancer cell death occurring through necrotic, apoptotic, and autophagic pathways. There is increasing evidence that reovirus-induced antitumor immunity involving both innate and adaptive responses also contributes to therapeutic efficacy though this discussion is beyond the scope of this article. Here, we review our current understanding of the mechanism of oncolysis contributing to the broad anticancer activity of reovirus. Further understanding of reovirus oncolysis is critical in enhancing the clinical development and efficacy of reovirus.

## Introduction

### Reovirus structure

Reovirus is a member of the *Reoviridae* family of viruses whose name was coined in 1959 and derived from the fact that it is commonly isolated from the *r*espiratory and *e*nteric tract without an association with clinical symptoms, or an *o*rphan virus, although infection can be associated with mild respiratory and enteric symptoms in humans (1–5). Antibody neutralization and hemagglutination-inhibition studies have identified three distinct serotypes of reovirus [Type 1 Lang, Type 2 Jones, Type 3 Abney, and Type 3 Dearing (T3D)] (1, 3, 5).

**Figure 1:**
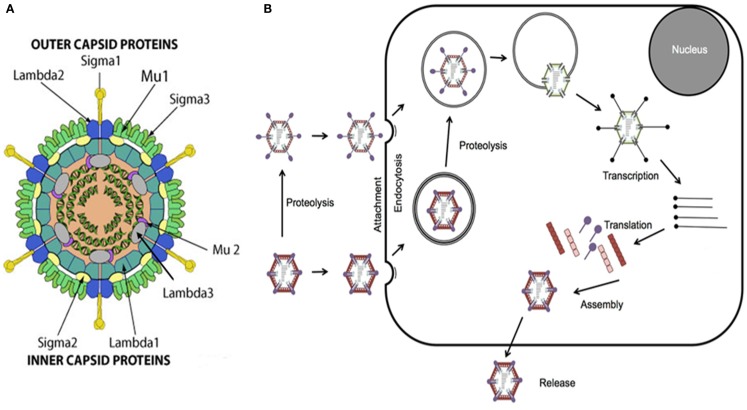
**Reovirus structure and infectious cycle**. **(A)** Reovirus is a non-enveloped double-stranded RNA (dsRNA) virus approximately 80 nm in diameter. The viral genome, consisting of 10 segments of dsRNA, is contained within an outer and inner capsid and encodes for structural proteins comprising the outer capsid including sigma 1 (σ1), sigma 3 (σ3), lambda 2 (λ2), and mu 1 (μ1), structural proteins comprising the inner capsid including sigma 2 (σ2), lambda 1 (λ1), lambda 3 (λ3), and mu 2 (μ2), and non-structural proteins including sigma 1s (σ1s), sigma NS (σNS), mu NS (μNS), and mu NSC (μNSC). σ1 has been identified as the viral attachment protein, μ1, μ2, and λ3 serve roles in viral replication, σ1s and σ3 appear to have roles in virulence, and σNS, μNS, and μNSC appear to be involved in the formation of viral inclusions. Figure reproduced with permission from ViralZone, SIB Swiss Institute of Bioinformatics. **(B)** Viral attachment to host cell surface glycans results in internalization of reovirus via receptor-mediated endocytosis. Alternatively, infectious subvirion particles (ISVPs) can be formed from proteolysis by extracellular proteases allowing their direct entry into cells via membrane penetration. Once internalized, the virus is transported to early and late endosomes where it undergoes proteolytic disassembly and degradation resulting in the formation of ISVPs and subsequently in the release of transcriptionally active viral core particles into the cytoplasm. Activated RNA-dependent RNA polymerase begins primary transcription within the core particles resulting in the release of primary transcripts that, along with protein products of early translation, form complexes or inclusions where further transcription and translation occur which, in turn, ultimately lead to viral replication and assembly, host cell death, and progeny release.

Reovirus is a non-enveloped double-stranded RNA (dsRNA) virus comprised of an outer and inner protein shell which altogether form a 20-sided icosahedral capsid (1, 2, 4, 6). The entire virus has an approximate diameter of 80 nm and houses its genome consisting of 10 segments of dsRNA that encode for structural and non-structural proteins involved in viral attachment, viral replication, virulence, and formation of viral inclusions (Figure 1) (1, 4, 7–10).

### Reovirus infectious cycle

The infectious life cycle of reovirus begins with attachment of viral protein sigma 1 (σ1) to target cell surface sialic acid residues, which have been shown to be α-linked 5-*N*-acetylneuraminic acid (Neu5Ac) and ganglioside GM2 glycan for serotypes T3D and Type 1 Lang, respectively (8, 11–13). Attachment to cell surface glycans facilitates binding to junctional adhesion molecule-A (JAM-A) which, in turn, results in internalization of reovirus via receptor beta 1 (β-1)-integrin mediated endocytosis (14, 15). Once internalized, the virus is transported to early and late endosomes where it undergoes proteolytic disassembly and degradation of the outer shell proteins sigma 3 (σ3) and mu 1 (μ1), in particular, by cysteine cathepsin proteases resulting in the formation of infectious subvirion particles (ISVPs) and ultimately in the release of transcriptionally active viral core particles, mediated by cleavage fragments of viral capsid proteins, into the cytoplasm (16–20). Of note, functional microtubules appear to be required, in part, for the process of endocytic sorting following internalization (21). ISVPs may also be formed from proteolysis by extracellular proteases, such as those in the gastrointestinal tract, allowing their direct entry into cells via membrane penetration (4, 22).

The viral core particles contain the necessary machinery including RNA-dependent RNA polymerase, guanylyltransferase, and methyltransferase to initiate viral replication (1, 2, 4–7). Activated RNA-dependent RNA polymerase begins primary transcription within the core particles resulting in the release of primary transcripts that, along with protein products of early translation, form complexes or inclusions where further transcription and translation occur which, in turn, ultimately lead to viral replication and assembly, host cell death, and progeny release (1, 2, 4–7). The events following virus internalization, endocytic processing, and viral core release remain poorly understood (Figure 1).

## Mechanism of Oncolysis

### Early investigations

The potential for wild-type reovirus as an anticancer agent was identified more than 30 years ago when studies demonstrated the preferential replication of reovirus in transformed cell lines but not in normal cells (23, 24). The mechanism of oncolysis by reovirus remained largely unknown until murine cell lines transfected with genes encoding the epidermal growth factor receptor (EGFR) demonstrated increased susceptibility to reovirus infection (25).

Normally, ligand-binding to EGFR activates the tyrosine kinase activity of EGFR and results in the autophosphorylation of the receptor’s cytoplasmic domain leading to the recruitment of phosphotyrosine-binding adaptor molecules such as Shc and Grb2 (26). Grb2, with or without association with Shc, recruits the guanine nucleotide exchange factor (GEF) son of sevenless (SOS) to the plasma membrane where it activates the small G protein Ras by promoting the exchange of GTP for GDP on Ras and therefore converting it from an inactivated GDP-bound state to an activated GTP-bound state (Ras-GTP) (26). Ras-GTP can then subsequently activate numerous downstream signaling pathways involved in cellular differentiation and proliferation (26). An example of one such pathway, which has been well characterized, involves Ras-GTP association with Raf-1 that activates the kinase activity of Raf resulting in the phosphorylation and activation of mitogen-activated protein kinase (MAPK) called MEK1 and MEK2 (26). Activated MEKs, in turn, phosphorylate and activate extracellular signal regulated kinases (ERKs) that ultimately translocate to the nucleus and partake in a number of pathways involved in cellular processes including transformation (26).

Interestingly, cell lines naturally resistant to reovirus infection demonstrated enhanced susceptibility when transformed with the v-*erbB* oncogene, which encodes for a truncated form of the EGFR lacking the extracellular ligand-binding domain but possessing constitutive tyrosine kinase activity, suggesting that reovirus infection is facilitated by EGFR-mediated pathways rather than binding of the virus to EGFR itself (27). Activating *Ras* mutations have been associated with approximately 30% of all human cancers though this number likely underestimates the true prevalence of activated Ras pathways in cancer given that mutations upstream and downstream of the Ras signaling pathway have also been associated with cellular transformation (28, 29). Not surprisingly, subsequent studies investigated the facilitation of reovirus infection by activated downstream EGFR-mediated pathways and, in particular, the activated Ras signaling pathway to uncover potential relationships to the mechanism of selective oncolysis.

### Activated Ras signaling pathway

Indeed, the downstream targets or intermediates to such pathways were elucidated when NIH-3T3 fibroblasts transfected with constitutively activated *SOS* or *Ras* oncogenes resulted in enhanced susceptibility to reovirus infection (30). Importantly, this finding occurred only in the presence of a zinc-inducible promoter, ZnSO_4_, which suggested that the activated Ras protein itself, rather than the effects of prolonged transformation, was sufficient to confer sensitivity to reovirus infection (30). Ras-GTP, however, is known to stimulate over more than 18 downstream effectors with the best characterized being the Raf kinases, the phosphatidylinositol 3-kinase (PI3K), and the GEFs for the small G protein Ral pathways (RalGEF) (29). Later studies aimed to narrow the list of potential downstream effectors of Ras important to reovirus oncolysis (31).

**Figure 2:**
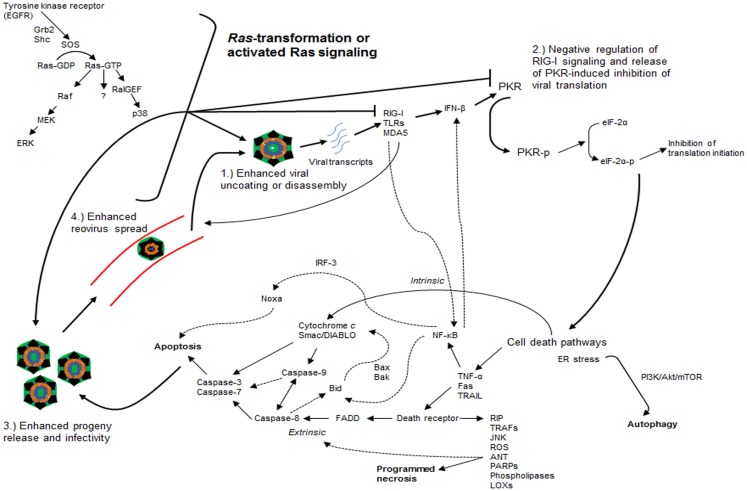
***Ras*-transformation affects several steps of the reovirus infectious cycle to promote oncolysis**. *Ras*-transformation affects multiple steps of the infectious life cycle in promoting reovirus oncolysis by: (1) enhancing virus uncoating and disassembly, (2) negative regulation of retinoic acid-inducible gene I (RIG-I) signaling and releasing dsRNA-activated protein kinase (PKR)-induced translational inhibition, (3) increasing progeny release through enhanced apoptosis and generating more infectious progeny, and (4) enhancing viral spread in subsequent rounds of infection. Reovirus-induced cancer cell death occurs through autophagic, apoptotic, and necrotic pathways. Programed necrosis or necroptosis occurs through binding of tumor necrosis factor-α (TNF-α), Fas ligand (Fas), and tumor necrosis factor-related apoptosis-inducing ligand (TRAIL) to death receptors leading to downstream signaling involving receptor interaction protein kinase (RIP) 1 and 3, cylindromatosis (CYLD), TNF receptor-associated factors (TRAFs), stress-activated c-Jun NH_2_-terminal protein kinase (JNK), reactive oxygen species (ROS), adenine nucleotide translocase (ANT), poly ADP-ribose polymerases (PARPs), phospholipases, and lipoxygenases (LOXs). Apoptosis occurs through both extrinsic [e.g., TRAIL binding to cell surface death receptor recruits Fas-associated death domain (FADD), which recruits and activates the initiator caspase-8 that ultimately activates effector caspases-3 and -7] and intrinsic pathways [cytochrome *c* and second mitochondrion-derived activator of caspase (Smac/DIABLO) release with activation of downstream effector caspases with or without caspase-9]. Autophagy occurs through endoplasmic reticulum (ER) stress and phosphatidylinositol 3-kinase (PI3K)/Akt/mammalian target of rapamycin (mTOR)-mediated signaling. Dashed arrows represent putative cross-talk and suggested signaling pathways. EGFR, epidermal growth factor receptor; SOS, son of sevenless; RalGEF, guanine nucleotide exchange factors (GEFs) for the small G protein Ral pathways; MEK, mitogen-activated protein kinase (MAPK) kinase; ERK, extracellular signal regulated kinase; TLRs: toll-like receptors; MDA5, melanoma differentiation-associated protein 5; IFN-β, interferon-beta; eIF-2α, eukaryotic initiation factor 2α; NF-κB, nuclear factor kappa light-chain enhancer of activated B cells; IRF-3, interferon regulatory factor 3.

When *Ras*-transformed NIH-3T3 fibroblasts expressing mutations in the effector-binding domains of the Ras protein were used, reovirus replication was found to be independent of signaling through Raf or PI3K downstream pathways (31). Only the V12G37 mutant, which retained RalGEF signaling capability, rendered *Ras*-transformed cells susceptible to reovirus infection while the use of a dominant-negative mutant of Ral rendered transformed cells non-permissive to reovirus infection (31). Interestingly, an activated mutant of RalGEF, Rlf, permitted reovirus replication in the absence of an activating *Ras* mutation (31). Lastly, it was demonstrated that p38 kinase (a downstream effector of RalGEF), but not stress-activated c-Jun NH_2_-terminal protein kinase (JNK), plays a role in establishing reovirus infection (31). In short, these findings implicate the Ras/RalGEF/p38 pathway in the promotion of selective reovirus replication (Figure 2) (31).

Early evidence suggested that increased expression of reovirus proteins was observed in *Ras*-transformed cells and correlated with elevated virus titers, while relatively impaired expression was observed in untransformed cells (29, 30). In untransformed cells, the inhibition of translation appeared to be a key step in preventing reovirus replication given that reovirus demonstrated equivalent binding, entry, and primary transcription in both *Ras*-transformed and untransformed cells (30, 31). A separate study highlighted the potential inhibitory activity of the reovirus structural protein σ3 on the dsRNA-activated protein kinase (PKR) (32). As a result, the role of PKR in reovirus oncolysis was implicated in lieu of the above observations (29).

In the presence of viral infection, viral transcripts are recognized and bound by receptors including toll-like receptors (TLRs), retinoic acid-inducible gene I (RIG-I), and melanoma differentiation-associated protein 5 (MDA5) resulting in the activation of transcription factors such as nuclear factor kappa light-chain enhancer of activated B cells (NF-κB) and interferon regulatory factor 3 (IRF-3) (33, 34). These factors then induce the release of type I interferons [interferon-beta and -alpha (IFN-β and -α)] that upregulate the expression of interferon-stimulated genes (ISGs), including the production of the serine/threonine kinase, PKR, involved in viral transcript degradation and inhibition of viral protein synthesis (33–35). PKR itself can bind to dsRNA resulting in dimerization, autophosphorylation, and activation (35). The same phenomenon has been observed in response to the presence of reovirus S1 mRNA as well (36). Activated PKR phosphorylates eukaryotic initiation factor 2α (eIF-2α) rendering it inactive and thereby leading to the inhibition of protein synthesis and viral replication (35).

It was determined that PKR remained inactivated in *Ras*-transformed cells thereby establishing the link between an activated Ras signaling pathway and PKR in reovirus oncolysis (30). The link between *Ras*-transformed cells and PKR inactivation has previously been suggested, but the use of a specific inhibitor to PKR phosphorylation by Strong et al. (30) that restored reovirus translation in untransformed cells offered evidence to the direct role PKR plays in determining resistance to selective reovirus replication (30, 37). Later studies demonstrated that *Ras*-transformation, through the MEK/ERK pathway, enhanced reovirus spread in subsequent rounds of infection by suppressing viral transcript-induced interferon-beta (IFN-β) production through negative regulation of RIG-I signaling (33). Accordingly, knockdown of either RIG-I or PKR led to increased susceptibility of untransformed cells to reovirus infection (33). The exact mechanism behind the coordination of *Ras*-transformation and PKR-mediated promotion of viral replication is unclear, but it remains among the best characterized processes in the understanding of reovirus oncolysis.

The coordination between the activated Ras signaling pathway, RIG-I and interferon signaling pathways, and PKR in the promotion of reovirus translation represents one potential mechanism among several emerging concepts detailing the effects of *Ras*-transformation on the reovirus infectious cycle (33, 38, 39). Other steps of the infectious cycle appear to be affected by *Ras*-transformation and involved in reovirus oncolysis as well. For example, in contrast to reovirus infection-susceptible glioma cell lines and *Ras*-transformed NIH-3T3 fibroblasts, reovirus-resistant and untransformed cells demonstrated prohibition of viral disassembly or uncoating due to the lack of degradation of σ3 and cleavage of μ1 proteins indicative of ISVP formation (38). Interestingly, disassembly restrictive cells introduced to ISVPs (*in vitro*) or grown as a tumor (*in vivo*), where elevated levels of active cathepsin B and L were observed in tumors, demonstrated productive reovirus infection and highlighted the significance of reovirus uncoating to susceptibility to oncolysis (38). Indeed, *Ras*-transformation of fibroblastic cell lines is associated with increased levels of cathepsin proteases (40, 41).

A separate study corroborated these findings when *Ras*-transformed NIH-3T3 fibroblasts demonstrated a threefold enhancement in reovirus uncoating compared to untransformed cells (39). Importantly, reovirus particles purified from *Ras*-transformed cells were four times more infectious than those from untransformed cells, and progeny release, mediated by caspase-induced apoptosis, was nine times more efficient in *Ras*-transformed cells when compared to untransformed cells (39). Of note, reovirus-induced apoptosis appears to be regulated, in part, through JNK signaling though the complexities and host of mediators involved in apoptosis by reovirus will be discussed later (42).

Altogether, these studies highlight two important points regarding reovirus oncolysis: (1) although prior studies implicate the Ras/RalGEF/p38 pathway in the promotion of reovirus oncolysis independent of Raf and JNK signaling, MEK/ERK, and JNK appear to have roles in reovirus infectivity and reovirus-induced apoptosis, and (2) further understanding of the reovirus infectious cycle has demonstrated that *Ras*-transformation affects multiple steps of the cycle, in addition to viral translation, such as viral uncoating or disassembly, generation of viral progeny with enhanced infectivity, release of progeny through enhanced apoptosis, and viral spread in subsequent rounds of infection (Figure 2) (31, 33, 38–42).

### Pathways of reovirus-induced cell death

There remains great debate in the pathway(s) by which reovirus induces cancer cell death. On one hand, intratumoral injections of reovirus into mouse xenograft models of human head and neck squamous cell carcinoma (SCC) induced tumor cell death through overwhelming burden of viral replication or cell lysis as evidenced by significant necrosis in the absence of apoptosis in pathologic specimens (43). Indeed, necrotic cell death appears to be a regulated process, more so than once believed, as programed necrosis or necroptosis is induced by binding of tumor necrosis factor-α (TNF-α), Fas ligand (FasL), and tumor necrosis factor-related apoptosis-inducing ligand (TRAIL) to death receptors leading to downstream signaling involving mediators such as receptor interaction protein kinase (RIP) 1 and 3, cylindromatosis (CYLD), TNF receptor-associated factors (TRAFs), JNK, reactive oxygen species (ROS), adenine nucleotide translocase (ANT), poly ADP-ribose polymerases (PARPs), phospholipases, and lipoxygenases (LOXs, Figure 2) (44–46).

On the other hand, apoptosis is a firmly established means of reovirus-induced cell death and has been demonstrated to be caspase-dependent and enhanced by activated Ras signaling (39, 47). Reovirus-induced apoptosis proceeds through both death receptor-associated (extrinsic) and mitochondrial-associated (intrinsic) pathways (48). For example, binding of the ligand TRAIL to the TNF superfamily of cell surface death receptors following reovirus infection recruits the adaptor molecule, Fas-associated death domain (FADD), which recruits and activates the initiator caspase-8 that ultimately activates effector caspases-3 and -7, forming part of the final common pathway for both extrinsic and intrinsic pathways (Figure 2) (48–50). In addition, following reovirus infection, the mitochondrial pro-apoptotic proteins cytochrome *c* and second mitochondrion-derived activator of caspase (Smac/DIABLO) are released, without disturbance of the mitochondrial membrane potential or release of apoptosis-inducing factor (AIF), and eventually activate downstream effector caspases with or without caspase-9 (caspase-9 is activated by cytochrome *c* but has been shown to be dispensable in the mitochondrial-associated pathway) (48, 51). There is a great degree of cross-talk between both apoptotic pathways, for example, activation of caspase-8 in the extrinsic pathway leads to the cleavage of the pro-apoptotic protein Bid and results in the release of cytochrome *c* and Smac/DIABLO (presumably through pro-apoptotic proteins Bax or Bak), activation of caspase-9, and eventual activation of effector caspases (Figure 2) (48, 51).

As previously suggested, viral uncoating or disassembly is critical for reovirus-induced apoptosis (52). Studies have identified several key components of the viral structure implicated in apoptosis including σ1, sigma 1s (σ1s), and μ1 in association with σ3 (53, 54). Ectopic expression of μ1 activated both extrinsic and intrinsic apoptotic pathways characterized by activation of initiator caspases-8 and -9, release of cytochrome *c* and Smac/DIABLO into the cytosol, and activation of downstream effector caspase-3 independent of the pro-apoptotic B-cell lymphoma-2 (Bcl-2) family members Bax and Bak (55).

The complexity of reovirus-induced apoptosis continues to grow (Figure 2). The roles of TRAIL, Bid, Bax, and Bak in reovirus-induced apoptosis have previously been discussed (48–51). As previously mentioned, JNK also regulates reovirus-induced cell death as activation of caspase-3 and apoptosis were inhibited by cells deficient with MEK kinase 1, an upstream activator of JNK (42). NF-κB also appears to play crucial roles in reovirus-induced apoptosis via stabilization of the tumor suppressor p53 and NF-κB-dependent mechanisms leading to Bid cleavage (56–58). Apoptosis by reovirus appears to require IRF-3 and NF-κB, in part, for efficient expression of the pro-apoptotic member of the Bcl-2 family, Noxa, independent of IFN-β induction (34). Reovirus appears to downregulate cellular FLICE inhibitory protein (cFLIP) and Akt activation to increase susceptibility to TRAIL-induced apoptosis in ovarian cancer cells and gastric cancer cells, respectively (59, 60). In neurons, reovirus-induced apoptosis is facilitated by upregulation of death-associated protein 6 (Daxx), an adaptor between the Fas death receptor and JNK signaling cascade, within the cytoplasm (61, 62). Moreover, apoptosis appears to be facilitated by reovirus-induced inhibition of microRNA-let-7d and upregulation of caspase-3 activity (63).

Alternatively, autophagy appears to be yet another mechanism of reovirus-induced cell death as reovirus infection of multiple myeloma cells demonstrated oncolysis mediated by both apoptotic and autophagic pathways (64, 65). Specifically, autophagy, as detected by Cyto-ID staining and vesicle colocalization with LC3-II (a marker of autophagosomes), was evident in human multiple myeloma cells (RPMI 8226) at 24 and 48 h of reovirus treatment (64). A fourfold reduction in autophagy by 48 h of reovirus infection was demonstrated when RPMI 8226 cells were pre-treated with the autophagy inhibitor 3-methyladenine (3-MA) (64). Understanding of the mechanisms behind mammalian reovirus-induced autophagy is limited to the above studies; however, *in vitro* studies involving avian reovirus (ARV) have provided further insight (66, 67). ARV infection of Vero cells *in vitro* induced autophagy via PI3K/Akt/mammalian target of rapamycin (mTOR) signaling detected by immunoblotting (67). Furthermore, pre-treatment of primary chicken fibroblasts and Vero cells with rapamycin, an mTOR inhibitor known to induce autophagy, and chloroquine, an inhibitor of lysosome–autophagosome fusion and autophagy, resulted in increased and decreased viral production, respectively, in ARV-infected cells (67). Separate studies have highlighted that endoplasmic reticulum (ER) stress not rescuable by the unfolded protein response (UPR) has been shown to enhance autophagy via negative regulation of the Akt/tuberous sclerosis protein (TSC)/mTOR pathway (68). Interestingly, reovirus-mediated apoptosis of multiple myeloma cells is also characterized by the stimulation of ER stress along with induction of Noxa (69). Indeed, growing evidence suggests autophagy mediated by PI3K/Akt/mTOR signaling and ER stress as a potential mechanism of reovirus-induced cancer cell death, although further investigation is warranted.

Lastly, it should be noted that reovirus-induced cell death in *Ras*-transformed cells is not absolute, but rather enhanced or more efficient relative to untransformed cells (29, 30, 39). The cytopathicity of reovirus infection in normal cells has long been characterized by respiratory and enteric pathogenicity in humans with seropositivity having been documented in as many as 70–100% of subjects (1–5). In clinical trials involving reovirus monotherapy in the treatment of cancer, common treatment-related adverse effects have included nausea, vomiting, fatigue, fever, myalgias, and other constitutional symptoms consistent with its relatively mild and benign viral pathophysiology in humans (1, 2, 4, 6, 7). The increased sensitivity for replication in cancer cells and excellent toxicity profile demonstrated in recent trials underscore the promising clinical potential of reovirus as an anticancer agent.

## Conclusion

Reovirus is a dsRNA virus whose mechanism of oncolysis remains unclear though activated Ras signaling, involving upstream and downstream mediators, appears important to permissiveness to reovirus replication. In promoting oncolysis, *Ras*-transformation affects multiple steps of the infectious life cycle including viral uncoating and disassembly, releasing PKR-induced translational inhibition, generation of viral progeny, release of progeny, and viral spread with reovirus-induced cancer cell death occurring through necrotic, apoptotic, and autophagic pathways. However, several studies have highlighted that reovirus oncolysis occurs independent of activated EGFR and Ras signaling pathways, while others have linked reovirus oncolysis to cell cycle phase (70–72). Undoubtedly, despite our progress in understanding reovirus oncolysis, further investigation into the mechanism of preferential replication in cancer cells is still warranted to enhance the antitumor efficacy of reovirus whose development has currently expanded to 32 clinical trials (both ongoing and completed) in the treatment of cancer.

## Conflict of Interest Statement

The authors declare that the research was conducted in the absence of any commercial or financial relationships that could be construed as a potential conflict of interest.
